# Bronchopulmonary dysplasia in extremely premature infant with congenital lobar emphysema: a case report

**DOI:** 10.1186/s12887-021-02772-3

**Published:** 2021-07-05

**Authors:** Bingchun Lin, Huitao Li, Chuanzhong Yang

**Affiliations:** Department of Neonatology, Shenzhen Maternity & Child Healthcare Hospital, Cheeloo College of Medicine, Shandong University, Shenzhen, Guangdong China

**Keywords:** Congenital lobar emphysema, Extremely premature infants, Bronchopulmonary dysplasia

## Abstract

**Background:**

Congenital lobar emphysema (CLE) is a congenital pulmonary cystic disease, characterized by overinflation of the pulmonary lobe and compression of the surrounding areas. Most patients with symptoms need an urgent surgical intervention. Caution and alertness for CLE is required in cases of local emphysema on chest X-ray images of extremely premature infants with bronchopulmonary dysplasia (BPD).

**Case presentation:**

Here, we report a case of premature infant with 27 + 4 weeks of gestational age who suddenly presented with severe respiratory distress at 60 days after birth. Chest X-ray and computed tomography (CT) indicated emphysema in the middle lobe of the right lung. The diagnosis of CLE was confirmed by histopathological examinations.

**Conclusions:**

Although extremely premature infants have high-risk factors of bronchopulmonary dysplasia due to their small gestational age, alertness for CLE is necessary if local emphysema is present. Timely pulmonary CT scan and surgical interventions should be performed to avoid the delay of the diagnosis and treatment.

**Supplementary Information:**

The online version contains supplementary material available at 10.1186/s12887-021-02772-3.

## Background

Congenital lobular emphysema (CLE) is a rare lung malformation, with an incidence of 1/20,000–30,000 live births [1, 2]. It is characterized by overinflation of the pulmonary lobes and compression of the surrounding areas. The most common affected lobe is the left upper lobe, followed by the right upper lobe and the right middle lobe [3]. Usually, a single lobe is involved, whereas the involvement of multiple or bilateral lobes is rare. The etiology of this malformation is still unknown. It may be caused by the partial obstruction of the bronchus, leading to pressure symptoms in the adjacent organs [4]. Due to the limited and insufficient understanding of the disease, the mortality rates of patients have been high because of misdiagnosis or mistreatment. When local emphysema is present on the chest X-ray images of extremely premature infants with bronchopulmonary dysplasia (BPD), timely pulmonary CT scan should be performed to avoid misdiagnosis in case of an unstable infant’s respiratory status. Here, we report a case of extremely premature infant of 27 + 4 weeks of gestational age with CLE in the middle lobe of the right lung. Detailed description has been provided below.

## Case presentation

**Fig. 1:**
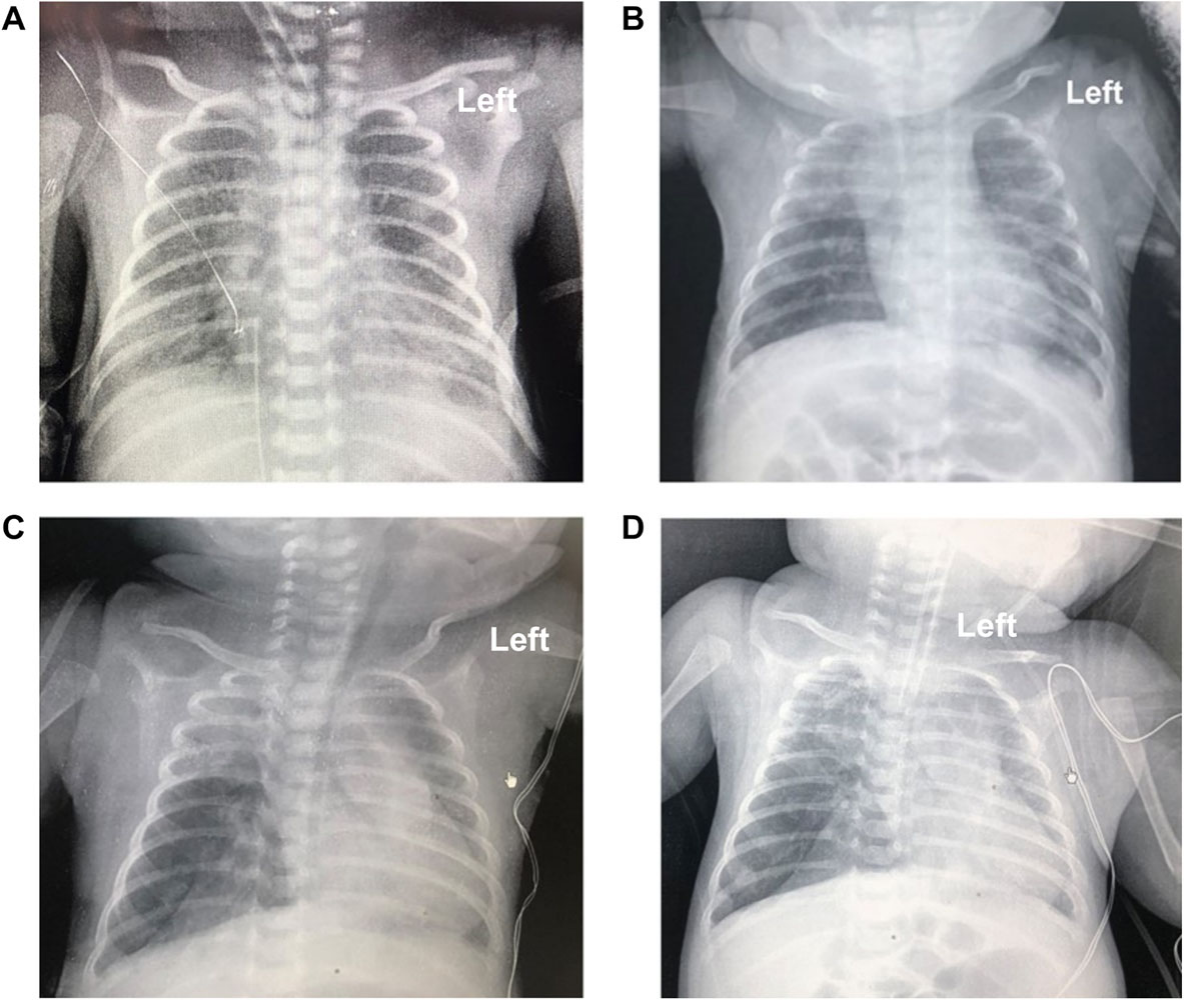
Chest radiography at D1, D10, D55, and D61. Chest X-ray revealed gradual progression of the mediastinal hernia

**Fig. 2:**
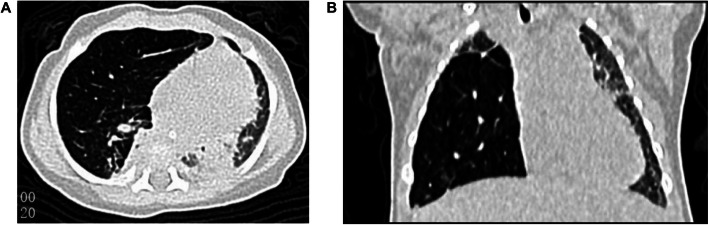
Chest CT at 73 days after birth revealed emphysema in the middle lobe of the right lung with mediastinal hernia

**Fig. 3:**
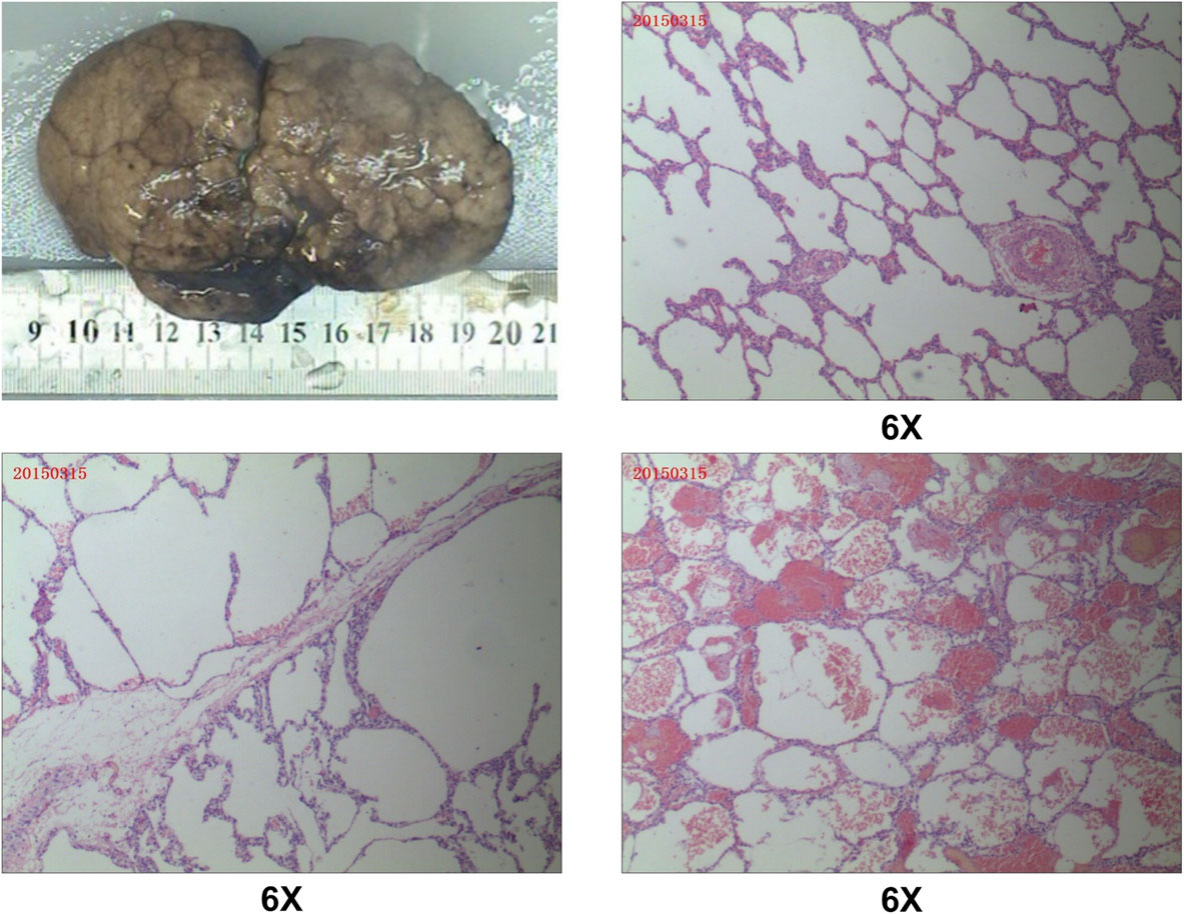
Pathological findings. Excised lung tissue showed lesions consistent with emphysema

Our case was of a female infant of 27 + 4 weeks of gestational age, G2P1, who was a test-tube baby. She was the first baby of twins in her family. On August 26, 2014, the baby was delivered naturally in Shenzhen Maternity & Child Healthcare Hospital (Shenzhen, Guangdong, China), with an Apgar score of 3 at 1 min, 4 at 5 min, and 7 at 10 min, and a birth weight of 1150 g. She was diagnosed as premature, extremely premature, very-low-birth-weight infant with neonatal pulmonary hyaline membrane disease (grade III). Placental histology examinations (Supplementary Fig. S1) and the blood test did not indicate fetal inflammation. After admission, she was administered with pulmonary surfactant through endotracheal intubation, mechanical ventilation for 2 days, non-invasive assisted ventilation and continuous positive airway pressure (CPAP) for 9 days, followed by low-flow oxygen delivery, intravenous nutrition, and other therapeutic measures. She was diagnosed with BPD at day 28 (D28) after birth for the oxygen requirement. At D60, her condition was stable, and only intermittent low-flow oxygen inhalation was needed. Soon afterwards, sudden severe respiratory distress occurred, with cyanosis and a decreased heart rate, and tracheal intubation was conducted for ventilation. Blood gas analysis results revealed carbon dioxide retention and a partial pressure of carbon dioxide (PCO_2_) fluctuating within 50–78 mmHg. Chest X-ray showed an increased density shadow in the right upper lung field and increased transmittance in the right middle lung field (Fig. 1). Chest CT revealed pneumonia with atelectasis in the upper and lower lobes of the right lung and emphysema in the middle lobe of the right lung with mediastinal hernia (Fig. 2), which were considered as congenital emphysema. Active anti-infection and mechanical ventilation therapy was applied. Corticosteroid treatment with three courses of dexamethasone was also conducted. The first course was given at D75 for 9 days, starting with a dose of 0.3 mg/kg/d; the second course was administered at D99 for 21 days, with an initial dose of 0.5 mg/kg/d, and the third course was implemented at D134 for 14 days and started with a dose of 1 mg/kg/d. However, no dyspnea relief was achieved; the blood gas analysis still showed a high level of PCO_2_. CT re-examination was still considered as congenital emphysema. Hence, we reviewed the chest radiograph at D10 which showed suspicious emphysema in the middle lobe of the right lung and the chest radiograph at D55 which revealed obvious emphysema in the middle lobe of the right Lung. On January 26, 2015, the patient was transferred to Shenzhen Children’s Hospital for surgical exploration, which suggested congenital lobar emphysema (of the right middle lobe). The pathological results of the excised lung tissue showed lesions consistent with emphysema, without obvious arrested alveolarization and decreased secondary crest (Fig. 3). After surgery, the patient’s ventilator and oxygen dependence were rapidly relieved, and she was cured and discharged 28 days postoperatively. After discharge, the infant was followed up for 5 years. She had good growth and development, with no recurrent respiratory tract infection.

## Discussion and conclusions

Congenital lobar emphysema is defined as the hyperinflation of one or more pulmonary lobes due to the partial obstruction of the bronchus, which causes pressure symptoms in the adjacent organs. CLE is a congenital pulmonary malformation that can be detected by prenatal ultrasound at 18–20 weeks of gestation. However different prenatal diagnosis rates of this disease have been reported in the literature. The prenatal diagnosis rate of congenital pulmonary cystic disease in 28 cases studied by He et al. was only 14.29% [5], whereas that of congenital pulmonary cystic adenomatoid malformation reported by Hong et al. was 87.5% [6]. This condition is more common in males, with a male to female ratio of 3:1 [7]. CLE is one of the rarest causes of respiratory distress in newborns [8]. The clinical manifestations of CLE after birth depend on the degree of the pulmonary lobe expansion and the compression exerted on adjacent tissues. Notably, in an earlier study, 33% of the patients had their symptoms at birth, 50% in the neonatal period, but most patients experienced them within 1 year of age [9]. Generally, patients have no prodromal infection. Nevertheless, dyspnea and other symptoms occur rapidly, which may endanger life. The chest X-ray could show emphysema of the affected lobes, with atelectasis in the adjacent lobes. In the early-newborn stage, due to poor pulmonary fluid drainage, no abnormal finding was established in the chest radiograph at D1, and the over-bright dilated pulmonary lobes appeared after several days. Notably, however, CT scan may reveal abnormalities. Patients with respiratory symptoms in the neonatal period should not be treated conservatively, and surgery should be performed as soon as possible once the diagnosis has been confirmed [10]. The early understanding of this disease was insufficient, and patients often died due to misdiagnosis and mistreatment. Nonetheless, in the past 20 years, the diagnosis and treatment of this disease have been significantly improved. However, misdiagnosis in neonates in whom the disease has not been detected before delivery is still easy to occur if they have sudden dyspnea. Many cases of CLE were reported to be misdiagnosed as pneumothorax and chest drainage was performed [11].

In recent years, more and more extremely premature infants have survived, with a high incidence of BPD. In cases of dyspnea accompanied by CLE, it is likely that patients could be treated for severe BPD, similarly to the case of this infant, resulting in delay of proper therapy. In this case, no CLE was detected before delivery, and pulmonary lobar emphysema was not found in the early postnatal chest radiograph, and thus the disease was not identified early. Two months after birth, the patient had sudden dyspnea and needed ventilation. After the application of treatment for severe BPD, no significant improvement was noted, but the disease was established by a timely pulmonary CT examination. In general, the lung injures caused by BPD are mainly diffuse lesions. In the early postnatal period, severe BPD is rarely manifested as obvious emphysema and atelectasis. In addition, this infant had no long-term history of mechanical ventilation or hyperoxia, hyperventilation therapy in the early stage. Hence, she did not have many high-risk factors for severe BPD. Therefore, in the present case, severe BPD should not have been considered to have caused dyspnea. The postoperative and follow-up results also indicated that the patient had non-severe BPD, and the lung function was good, except in the emphysematous lobes. The lung injures caused by BPD could not be improved quickly by surgery. As the pathological findings showed lesions consistent with emphysema without obvious arrested alveolarization and decreased secondary crest, we considered the main cause of the pulmonary emphysema was congenital. Therefore, a gene test could have provided valuable information in this situation.

Currently, few cases of extremely premature infants aged less than 28 weeks with BPD and CLE have been reported. The local emphysema present in such patients should increase the caution and alertness for CLE. Chest radiography alone is not sufficient, and the timely pulmonary CT examination is critical for proper diagnosis. Once the diagnosis has been confirmed, surgery should be urgently performed.

## Supplementary Information

**Additional file 1: Fig. S1** Pathological images.

## Data Availability

The datasets used and analyzed during the current study are available from the corresponding author on reasonable request.

## Abbreviations

CLE: Congenital lobar emphysema
CT: Computed tomography
BPD: Bronchopulmonary dysplasia
CPAP: Continuous positive airway pressure
PCO_2_: Partial pressure of carbon dioxide
