# Supplementary figures and images for: Bronchopulmonary dysplasia in extremely premature infant with congenital lobar emphysema: a case report
Source: BMC Pediatr. 2021 Jul 5;21:300. doi: 10.1186/s12887-021-02772-3 (PMC8256543; doi:10.1186/s12887-021-02772-3)

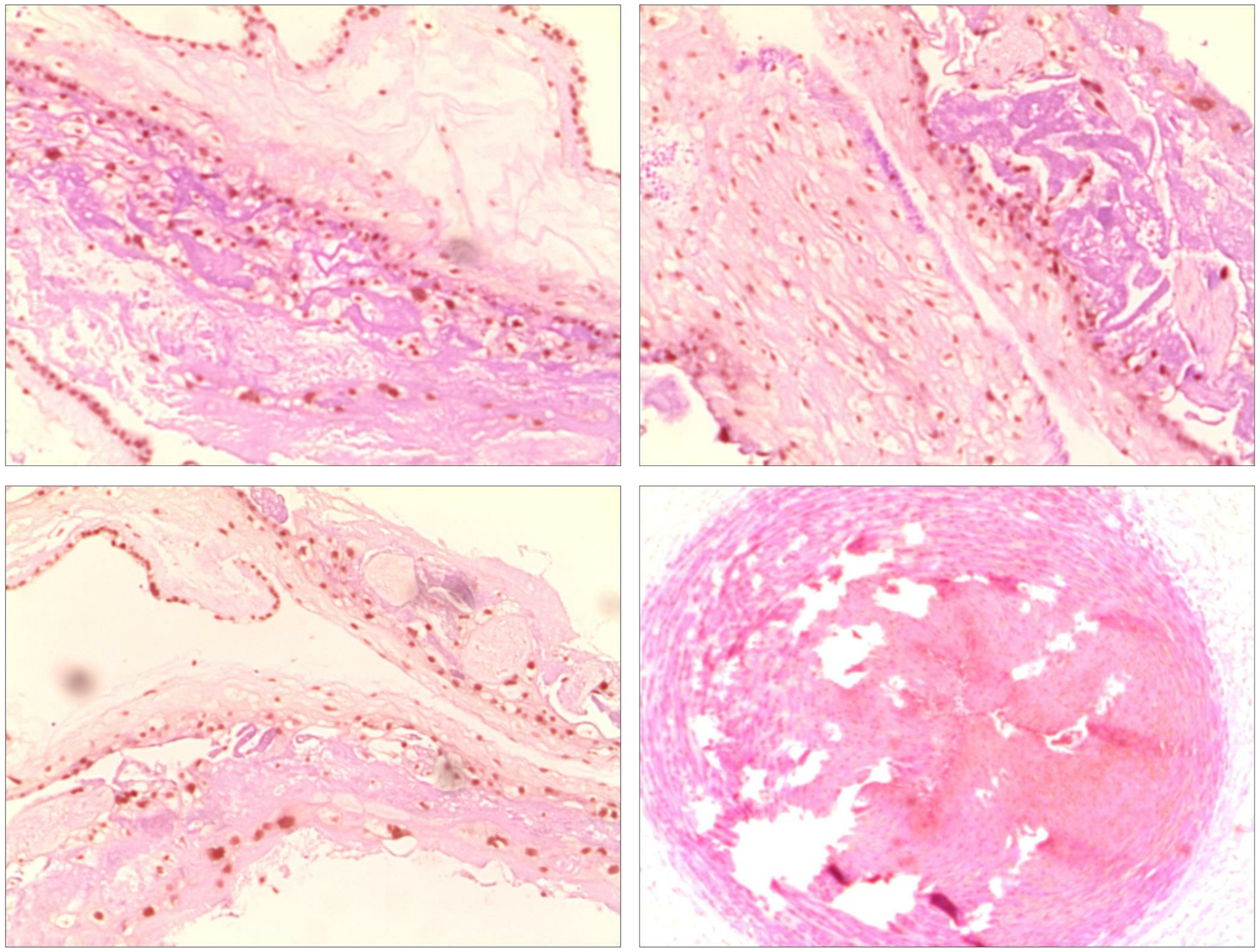

Supplement: Supplementary file 1 — Additional file 1: Fig. S1 Pathological images. [file 12887_2021_2772_MOESM1_ESM.tif]
